# Carbapenem-Resistant Citrobacter freundii and Escherichia coli Harboring a Common IncC-Type Plasmid Encoding IS*26* Upstream of *bla*_NDM-1,_ *sul1*, *aph(3′)-VI*, and *qacE* Isolated from Edible *Mastacembelidae* Fish

**DOI:** 10.1128/mra.01344-22

**Published:** 2023-04-03

**Authors:** Tatsuya Nakayama, Takahiro Yamaguchi, Doan Tran Nguyen Minh, Oanh Nguyen Hoang, Hien Le Thi, Phong Ngo Thanh, Phuong Hoang Hoai, Michio Jinnai, Phuc Nguyen Do, Chinh Dang Van, Yuko Kumeda, Atsushi Hase

**Affiliations:** a Graduate School of Integrated Sciences for Life, Hiroshima University, Hiroshima, Japan; b Division of Microbiology, Osaka Institute of Public Health, Osaka City, Osaka, Japan; c Division of Food Microbiology, Institute of Public Health, Ho Chi Minh City, Vietnam; d Kanagawa Institute of Public Health, Chigasaki City, Kanagawa, Japan; e Research Center for Microorganism Control, Osaka Prefecture University, Sakai City, Osaka, Japan; f Faculty of Contemporary Human Life Science, Tezukayama University, Nara City, Nara, Japan; Montana State University

## Abstract

Carbapenem-resistant Citrobacter freundii CF20-4P-1 and Escherichia coli EC20-4B-2 were isolated from edible *Mastacembelidae* in Vietnam. We present the draft genome sequences, and the complete plasmid genome sequencing was also performed by hybrid assembly sequencing of Oxford Nanopore and Illumina. The 137-kbp plasmid encoding the assembled *bla*_NDM-1_ was detected in both strains.

## ANNOUNCEMENT

As antibiotic-resistant bacteria (ARB) have reportedly been found in imported foods, the spread of plasmid-mediated ARB through food products is a concern (1). Here, we report the complete genome sequences of carbapenem-resistant Citrobacter freundii and Escherichia coli harboring a common plasmid isolated from fresh edible river fish, *Mastacembelidae*, purchased in Ho Chi Minh City, Vietnam.

Bacterial isolation and identification were performed as previously described (2). Fresh *Mastacembelidae* fish were purchased from a retail market in Ho Chi Minh City, Vietnam; their gut contents (5 g) were mixed with buffered peptone water (BPW) (45 mL). After incubation at 37°C for 24 h, BPW (100 μL) was spread on CHROMagar ECC (CE) (CHROMagar, Paris, France) containing meropenem (0.25 g/mL) and incubated at 37°C for 24 h. Several colonies were selected, and isolated *Enterobacteriaceae* were investigated for antibiotic susceptibility using the disk diffusion method (3). Two meropenem-resistant *Enterobacteriaceae* were identified as putative C. freundii and E. coli by using colony phenotype and CHROMagar Orientation. After subculturing on CE agar at 37°C for 17 h, bacterial DNA was extracted using the DNeasy blood and tissue kit (Qiagen, Hilden, Germany) for next-generation sequencing using short-read sequencing and Genomic-Tip 100/G (Qiagen). The extracted DNA was analyzed using a Qubit double-stranded DNA (dsDNA) high-sensitivity (HS) assay kit (Thermo Fisher Scientific, Waltham, MA, USA). For short- and long-read sequencing library preparation, the QIAseq FX DNA library UDI kit (Qiagen) and rapid barcoding kit (Oxford Nanopore Technologies, Oxford, UK) were used, and sequencing was performed using Illumina HiSeq (Illumina, San Diego, USA) with a 2 × 150-bp paired-end protocol and MinION (Oxford) with flow cells (R9.4.1; Oxford). After we obtained short-read sequences, trimming and quality checks were conducted using fastp v0.20.0 (4). Short- and long-read quality checks were performed using fastqc v0.11.9 (4) and NanoFilt v2.8.0 (5). Guppy v5.0.11 was used as the base caller. A hybrid assembly of Illumina (for C. freundii and E. coli, total reads, 5,474,476 and 5,830,566; mean length after filtering, 2 × 147-bp reads [paired end]; total bases, 806.9 and 859.4 Mb; coverage, 105.1× and 51.7×, respectively) and MinION (total reads, 45,747 and 22,371; *N*_50_ values, 19,696 and 18,934 bp; total bases, 510.5 and 236.1 Mb, respectively) sequencing data was performed using Unicycler v0.5.0 (6). Default parameters were used for all software unless otherwise specified. The assembled whole-genome sequencing (WGS) was analyzed using MLST2.0 and MobileElementFinderv1.0.3. SpeciesFinder confirmed that C. freundii and E. coli were isolated, and ResFinder analysis showed *bla*_NDM-1_, *sul1*, *aph(3′)-VI*, and *qacE* were found in both bacterial plasmids (Table 1).

**TABLE 1:** Genome information of Citrobacter freundii CF20-4P-1 and Escherichia coli EC20-4B-2 isolated from *Masacembelidae* fish

Characteristic^*a*^	Data for:			
	Citrobacter freundii CF20-4P-1		Escherichia coli EC20-4B-2	
	Whole genome	Plasmid	Whole genome	Plasmid
Strain name	CF20-4P-1	pCF20-4P-1-2	EC20-4B-2	pEC20-4B-2-2
MLST or Inc type	ST499	A/C2	ST155	A/C2
Total length (bp)	5,451,738	137,080	4,943,131	137,080
No. of contigs	5	1	3	1
GC content (%)	51.70	51.30	50.70	51.30
*N*_50_ (bp)	4,907,659	137,080	4,645,627	137,080
No. of CDSs	5,202	174	4,618	174
No. of rRNAs	25	0	22	0
No. of tRNAs	84	0	89	0
No. of mobile genetic elements	45	5 (IS*26*, IS*As17*, IS*Ec52*)	39	5 (IS*26*, IS*As17*, IS*Ec52*)
Antibiotic resistance genes	*aph(3′)-VI*, *qacE*, *qnrB38*, *qnrB60*, *sul1*, *bla*_CMY-65_, *bla*_NDM-1_	*qacE*, *sul1*, *aph(3′)-VI*, *bla*_NDM-1_	*aph(6)-Id*, *aph(3″)-Ib*, *aph(3′)-VI*, *qnrS1*, *dfrA14*, *sul2*, *sul1*, *tet(A)*, *bla*_TEM-1B_, *bla*_NDM-1_, *qacE*, *floR*	*qacE*, *sul1*, *aph(3′)-VI*, *bla*_NDM-1_

a MLST, multilocus sequencing typing; CDS, coding DNA sequence.

**FIG 1:**
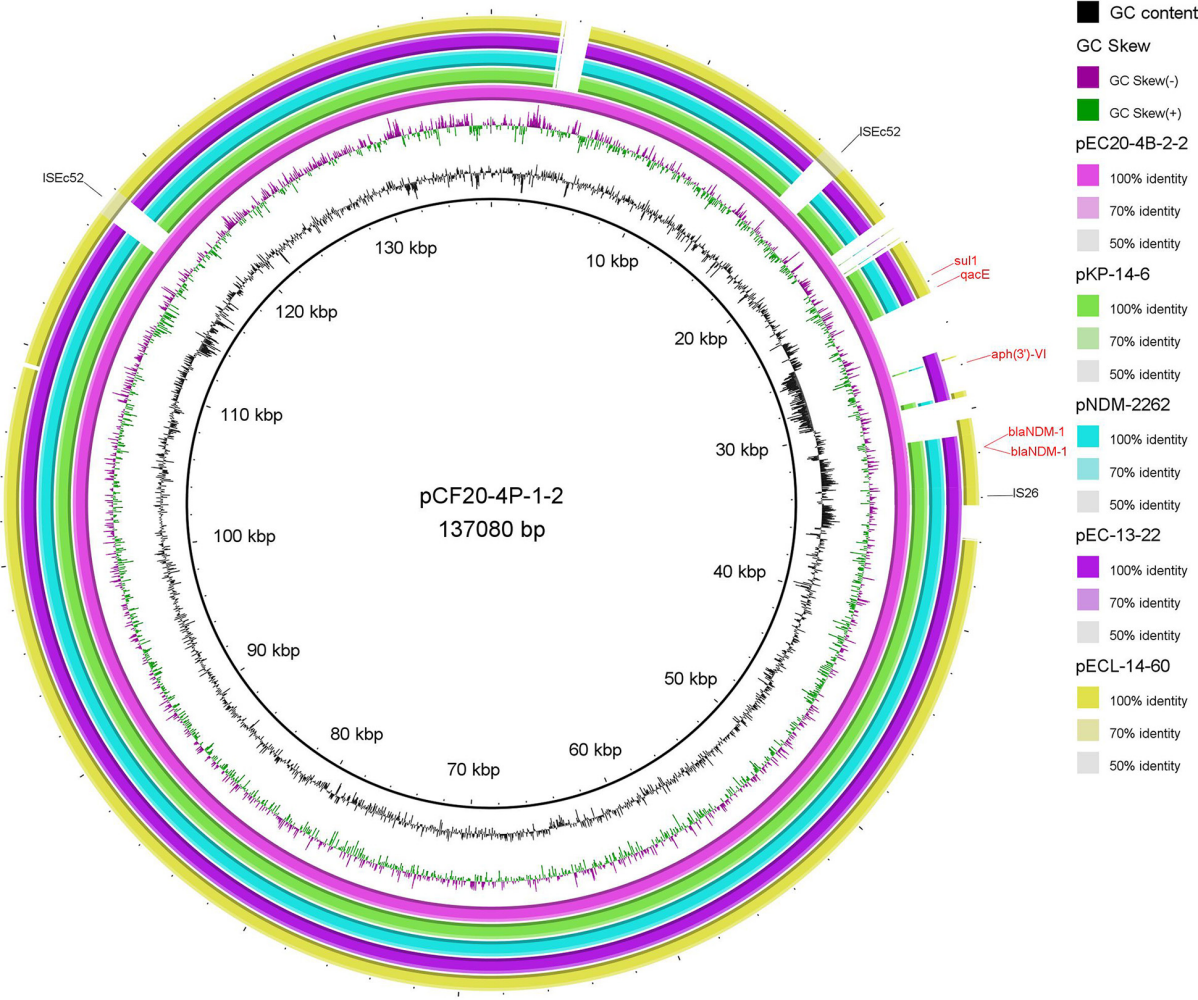
Complete plasmid map of C. freundii pCF20-4P-1-2 and E. coli pEC20-4B-2-2 isolated from shrimp. The complete genomes of four strains (GenBank accession no. MN175387, MH892479, MZ836796, and MZ836805) with high homology were selected. Plasmid maps were designed using BLAST Ring Image Generator v0.95.

A comparison showed that the plasmids carried by both bacteria were the same. The mobile genetic factors insertion sequences IS*26* and IS*Ec52* are located upstream and downstream of *bla*_NDM-1_, *qacE*, *sul1*, and *aph(3′)-VI* (Fig. 1). Therefore, this shared plasmid may cause horizontal transmission within *Enterobacteriaceae*.

### Data availability.

C. freundii CF20-4P-1 and E. coli EC20-4B-2 WGS, as well as pCF20-4P-1-2 and pEC20-4B-2-2 partial genome sequencing (PGS), were deposited in DDBJ and GenBank (accession numbers AP026939, AP026942, AP026937, and AP026940). The raw read SRA accession numbers are DRX382161, DRX382162, DRX382159, and DRX382160, with BioProject accession number PRJDB11927.
